# Salvianolic acid B ameliorates neuroinflammation and neuronal injury via blocking NLRP3 inflammasome and promoting SIRT1 in experimental subarachnoid hemorrhage

**DOI:** 10.3389/fimmu.2023.1159958

**Published:** 2023-07-26

**Authors:** Dayong Xia, Jinlong Yuan, Degang Wu, Haibin Dai, Zong Zhuang

**Affiliations:** ^1^ The Translational Research Institute for Neurological Disorders of Wannan Medical College, Department of Neurosurgery, the First Affiliated Hospital of Wannan Medical College (Yijishan Hospital of Wannan Medical College), Wuhu, China; ^2^ Department of Neurosurgery, Nanjing Drum Tower Hospital, Affiliated Hospital of Medical School, Nanjing University, Nanjing, China

**Keywords:** NLRP3, salvianolic acid B, subarachnoid hemorrhage, sirtuin 1, MCC950

## Abstract

The nucleotide-binding oligomerization domain (NOD)-like receptor family pyrin domain containing 3 (NLRP3) inflammasome-mediated immuno-inflammatory response plays a critical role in exacerbating early brain injury (EBI) after subarachnoid hemorrhage (SAH). Salvianolic acid B (SalB) has previously been shown to suppress neuroinflammatory responses in many disorders. Meanwhile, a previous study has demonstrated that SalB mitigated oxidative damage and neuronal degeneration in a prechiasmatic injection model of SAH. However, the therapeutic potential of SalB on immuno-inflammatory responses after SAH remains unclear. In the present study, we explored the therapeutic effects of SalB on neuroinflammatory responses in an endovascular perforation SAH model. We observed that SalB ameliorated SAH-induced functional deficits. Additionally, SalB significantly mitigated microglial activation, pro-inflammatory cytokines release, and neuronal injury. Mechanistically, SalB inhibited NLRP3 inflammasome activation and increased sirtuin 1 (SIRT1) expression after SAH. Administration of EX527, an inhibitor of SIRT1, abrogated the anti-inflammatory effects of SalB against SAH and further induced NLRP3 inflammasome activation. In contrast, MCC950, a potent and selective NLRP3 inflammasome inhibitor, reversed the detrimental effects of SIRT1 inhibition by EX527 on EBI. These results indicated that SalB effectively repressed neuroinflammatory responses and neuronal damage after SAH. The action of SalB appeared to be mediated by blocking NLRP3 inflammasome and promoting SIRT1 signaling.

## Introduction

Subarachnoid hemorrhage (SAH) causes devastating neurological damage, which seriously affects patients’ quality of life. Survivors still face a series of symptoms, including consciousness disturbance, physical impairment, mood disorders, and sleep disturbances (1). However, current treatments for SAH show low therapeutic efficacy. Evidence from experimental and clinical studies indicates that suppressing immuno-inflammatory responses could ameliorate neuronal injury and improve functional recovery after SAH (2–4). Thus, it is urgently needed to identify new treatment that targets immuno-inflammatory responses and SAH-related comorbidities.

It is becoming clear that dysregulation of inflammatory responses contributes greatly to the pathophysiological progress of early brain injury (EBI) after SAH (5, 6). Microglial over-activation has been implicated as a primary factor in inflammation-mediated brain damage (7, 8). After hemorrhage, proinflammatory microglia are rapidly activated and produce a variety of proinflammatory cytokines, which contribute to tissue injury and neurogenesis impairment. Therefore, repressing destructive inflammatory response might be a promising therapeutic approach for the treatment of SAH.

Salvianolic acid B (SalB), a polyphenolic compound isolated from *Salvia miltiorrhiza*, was developed as a potential treatment for cardiovascular and cerebral vascular diseases (9, 10). Increasing evidence has shown that SalB exhibits a broad range of pharmacological potentials, such as antioxidative, anti-inflammatory, anti-depression, and neuroprotective effects (11, 12). In central nervous system (CNS) diseases, SalB has been shown to protect against ischemic stroke, traumatic brain injury, vascular dementia, and spinal cord injury (13–15). A previous study also reported that SalB mitigated SAH-triggered oxidative damage by modulating sirtuin 1 (SIRT1) pathway (9). However, the therapeutic potential of SalB on immuno-inflammatory responses after SAH remains unclear. Accumulated evidence shows that SalB has a potent anti-inflammatory activity and is able to inhibit microglial activation in different CNS disorders (16, 17). Additionally, SalB has been reported to suppress nucleotide-binding oligomerization domain (NOD)-like receptor family pyrin domain containing 3 (NLRP3) inflammasome signaling, which plays a crucial role in microglia-mediated inflammatory responses after SAH (18, 19). Once activated, NLRP3 recruits the adapter apoptosis-related speck-like protein (ASC) and activates pro-caspase-1 (20). Caspase-1 cleaves the pro-inflammatory cytokines pro-interleukin (IL)-1β and IL-18 to execute the innate immune response and cell death (21, 22). Notably, inhibition of NLRP3 inflammasome activation could mitigate neuroinflammation and neurological impairment in animal models of SAH (23, 24). Thus, we hypothesized that SalB would ameliorate inflammatory brain injury and improve functional recovery after SAH. Mechanistically, we further confirmed whether the neuroprotective effects of SalB occur via inhibiting NLRP3 inflammasome and promoting SIRT1 pathway.

## Materials and methods

### Animals and SAH model

One hundred thirty-five adult male C57BL/6J mice (weighing 20-25 g) were employed in this study. All experimental procedures were approved by the Animal Ethics Review Committee of Yijishan Hospital. Establishing the SAH model induced by the endovascular perforation technique (25). In brief, after anesthetization with sodium pentobarbital (40 mg/kg), a sharpened 6-0 filament was advanced into the internal carotid artery and then progressed forward to puncture the junction of the right middle and anterior cerebral arteries. Sham-operated mice underwent the same procedure without perforating the cerebral artery.

### Experimental groups

A schematic of experimental protocols is given in **Supplementary Figure 1**. In the first set of experiments, mice were randomly divided into the sham (n = 6), SAH (n = 8, 2 mice died), SAH + 10 mg/kg SalB (n = 8, 2 mice died), SAH + 20 mg/kg SalB (n = 8, 2 mice died), and SAH + 40 mg/kg SalB (n = 7, 1 mice died) groups. The short-term and long-term functional behavior were evaluated.

The experimental design for the second experiment was to explore the potential role of SalB on neuroinflammation and its mechanisms after SAH. Mice were randomly divided into the sham (n = 12), SAH (n = 16, 4 mice died), and SAH + 20 mg/kg SalB (n = 15, 3 mice died) groups. Assessment methods included immunostaining, western blot, and enzyme-linked immunosorbent assay (ELISA).

In experiment 3, to validate the effects of SalB on NLRP3 inflammasome and SIRT1 signaling, mice were randomly divided into the SAH (n = 16, 4 mice died), SAH + 20 mg/kg SalB (n = 14, 2 mice died), SAH + 20 mg/kg SalB + EX527 (n = 16, 4 mice died), and SAH + 20 mg/kg SalB + EX527 + MCC950 (n = 15, 3 mice died) groups. Assessment methods included behavior tests, immunostaining, western blot, and ELISA.

### Behavioral analysis

A modified Garcia score was used to assess neurological deficits after SAH. The modified Garcia score is an 18-point scoring system, in which higher scores indicated better function (26). Beam balance test was performed to evaluate motor deficits. In brief, mice were placed on a beam (1-m length and 6-mm width) and their walking distance within 1 min was recorded. The cognitive impairment was assessed by Y-Maze test. Mice were placed on the center of the maze and allowed to move freely the apparatus for 5 min. The ratio of actual alternations to possible alternations was recorded as the spontaneous alternation performance.

### Pharmacological treatments

SalB (purity > 97%, Sigma-Aldrich) was dissolved in physiologic saline. Mice were administered SalB (10 mg/kg, 20 mg/kg, or 40 mg/kg, i.p.) starting 1 h after SAH and again every 24 h for 3 days. The SIRT1 inhibitor EX527 (10 mg/kg, Sigma-Aldrich) was prepared in 1% dimethyl sulfoxide (DMSO, Sigma-Aldrich) and administered intraperitoneally beginning 2 h before SAH operation. MCC950 (10 mg/kg, MedChem) was prepared in physiologic saline and administered intraperitoneally for 3 days before SAH construction. The doses of SalB, EX527, and MCC950 were chosen according to previous studies (9, 24, 27).

### ELISA

The levels of IL-1β, IL-6, and IL-18 in brain tissue were measured by using ELISA kits (EK201B2 for IL-1β, EK206HS for IL-6, EK218 for IL-18, Multi Sciences). In brief, diluted samples and cytokine standards were added to the coated 96-well plates. The optical density of each well was recorded. The concentrations of IL-1β, IL-6, and IL-18 were calculated according the standard curves, respectively.

### Western blotting

Brain tissues were lysed using RIPA supplemented with protease inhibitor (P2850, Sigma-Aldrich). A BCA Kit (P0012, Beyotime) was employed for protein quantification. Equal amounts of protein were loaded on SDS‐PAGE gels. After electrophoresis, they were transferred to polyvinylidene difluoride membranes. The membranes were blocked with 1% bovine serum albumin (BSA, ST2254, Beyotime) and then incubated with primary antibodies overnight at 4°C. The primary antibodies used for western blotting were shown as follows: anti-NLRP3 (ab263899, Abcam), anti-ASC (sc-22514, Santa Cruz), anti-caspase-1 (SC-56036, Santa Cruz), anti-c-caspase-1 (SC-398715, Santa Cruz), and anti-SIRT1(ab110304, Abcam). After being washed, they were incubated with corresponding secondary antibodies for 1 h. Protein signals were quantified using the Image J.

### Immunofluorescence staining

Mice were perfused with 4% paraformaldehyde (PFA, P0099, Beyotime). Their brains were fixed with 4% PFA for 48 h and then dehydrated into 30% sucrose. Brain sections (35 μm) were obtained by using a Leica CM1950 cryostat. Sections were incubated with primary antibodies overnight at 4°C. After washing, tissue samples were incubated for 2 h with corresponding secondary antibodies. And then, they were mounted with an antifade mounting medium with 4′,6-diamidino-2-phenylindole (DAPI, C1006, Beyotime). The primary antibodies used for immunofluorescence were shown as follows: anti-IL-1β (ab254360, Abcam), anti-CD68 (MCA1957GA, Bio-Rad), and anti-Iba1 (ab178847, Abcam). Pictures were acquired with a fluorescence microscope.

### TUNEL staining

The cell apoptosis was evaluated by using TUNEL *in situ* cell death detection kit (C1090, Beyotime). In brief, the sections were permeabilized with 0.5% Triton X-100 (9002-93-1, Thermo Scientific) and then blocked with 10% BSA. Then the tissue sections were incubated with the primary antibody against NeuN (MAB377, Millipore), and then incubated with TUNEL reaction mixture. After washing, they were mounted with an antifade mounting medium with DAPI. Pictures were acquired with a fluorescence microscope.

### Statistical analysis

GraphPad Prism 8.0 software was used for the statistical analysis. All data were expressed as the mean and standard deviation (SD). Comparison of means among multiple groups was performed using one-way or two-way ANOVA with Bonferroni *post hoc* test. A value of *P* < 0.05 was considered statistically significant.

## Results

### SalB improved short-term and long-term functional performance

**Figure 1:**
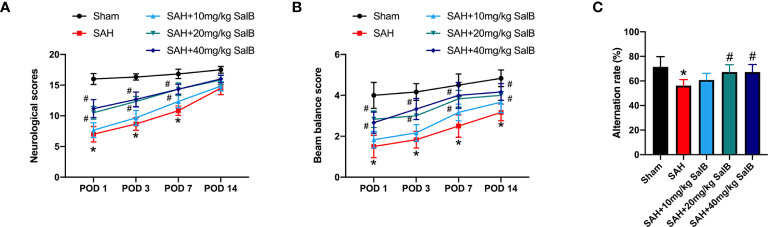
SalB improved short-term and long-term functional performance after SAH. **(A)** Neurological deficits test was performed on POD 1, 3, 7, and 14 (n = 6 mice/group). **(B)** Beam balance test was conducted on POD 1, 3, 7, and 14 (n = 6 mice/group). **(C)** SalB improved spatial working memory after SAH by the spontaneous alternation task in the Y-maze (n = 6 mice/group). Data are expressed as mean ± SD. *P < 0.05 vs Sham group; ^#^ *P* < 0.05 vs SAH group.

We first evaluated the effects of SalB on the short-term and long-term behavior function. It showed that administration of SalB at doses of 20 mg/kg and 40 mg/kg significantly mitigated neurological deficits score (**Figure 1A**) and improved motor performance (**Figure 1B**) at post-operative day (POD) 1, POD 3, and POD 7 when compared with the SAH group, whereas 10 mg/kg SalB did not improve behavior function (**Figures 1A, B**). Cognitive impairment after SAH is common and disabling. We further evaluated spatial working memory following SAH by using Y-Maze test. It showed that the SAH group exhibited more severe memory deficits in the spontaneous alternation task compared to the sham group, whereas SalB treatment at doses of 20 mg/kg and 40 mg/kg reduced the worse outcomes in the Y-maze task (**Figure 1C**). No significant differences between 20 mg/kg and 40 mg/kg SalB treatment on functional performance were detected. Thus, we used 20 mg/kg SalB for the remaining experiments.

### SalB reduced inflammatory response and microglial activation

**Figure 2:**
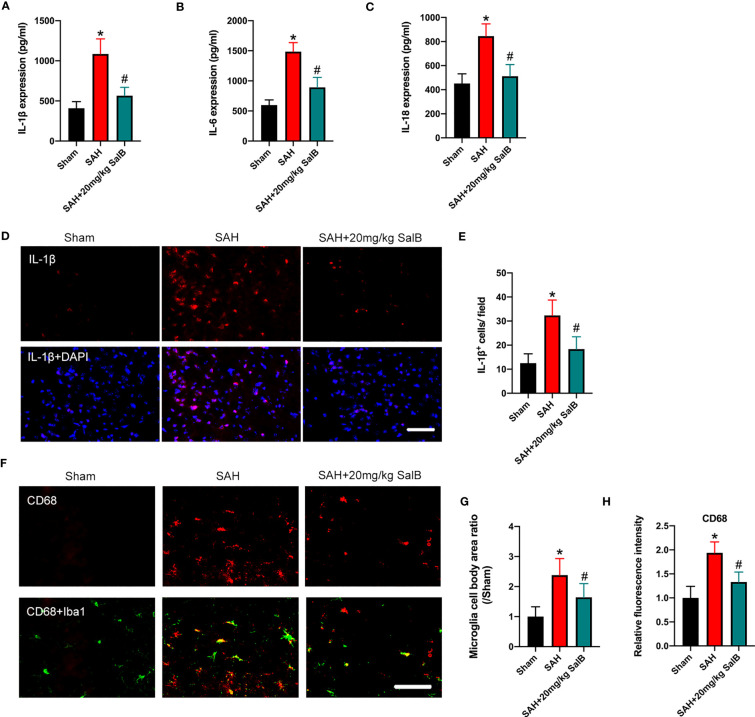
SalB reduced inflammatory response and microglial activation. Quantitative analysis of the levels of IL-1β **(A)**, IL-6 **(B)**, and IL-18 **(C)** by ELISA kits (n = 6 mice/group). **(D)** Representative immunofluorescent staining of IL-1β in brain cortex at 24 h after SAH. **(E)** Quantitative analysis of the number of IL-1β^+^ cells (n = 6 mice/group). **(F)** Representative immunofluorescent staining of Iba1 with CD68 in ipsilateral cortex. Quantitative analysis of the microglia cell body area ratio **(G)** and CD68 intensity **(H)** (n = 6 mice/group). Data are expressed as mean ± SD, scale bar = 50 μm. *P < 0.05 vs Sham group; ^#^ *P* < 0.05 vs SAH group.

Dysregulation of inflammatory responses contributes greatly to the pathophysiological progress of EBI after SAH. Microglial over-activation has been implicated as a primary factor in neuroinflammation (6). We then explored the effects of SalB on inflammatory responses and microglial activation. As shown in **Figure 2**, SAH insults induced a significant increase in pro-inflammatory cytokines release, including IL-1β (**Figure 2A**), IL-6 (**Figure 2B**), and IL-18 (**Figure 2C**) when compared with the sham group, whereas SalB markedly decreased these pro-inflammatory cytokines (**Figures 2A–C**). IL-1β immunofluorescence staining (**Figure 2D**) further confirmed that IL-1β expression in brain cortex was increased at 24 h after SAH, which could be significantly reduced after treatment with SalB (**Figure 2E**). Additionally, Iba1 and CD68 immunofluorescence staining (**Figure 2F**) further showed that SalB administration markedly suppressed microglial activation as evidenced by the decreased microglia cell body area (**Figure 2G**) and expression of CD68 (**Figure 2H**).

### SalB inhibited NLRP3 inflammasome signaling and increased SIRT1 expression

**Figure 3:**
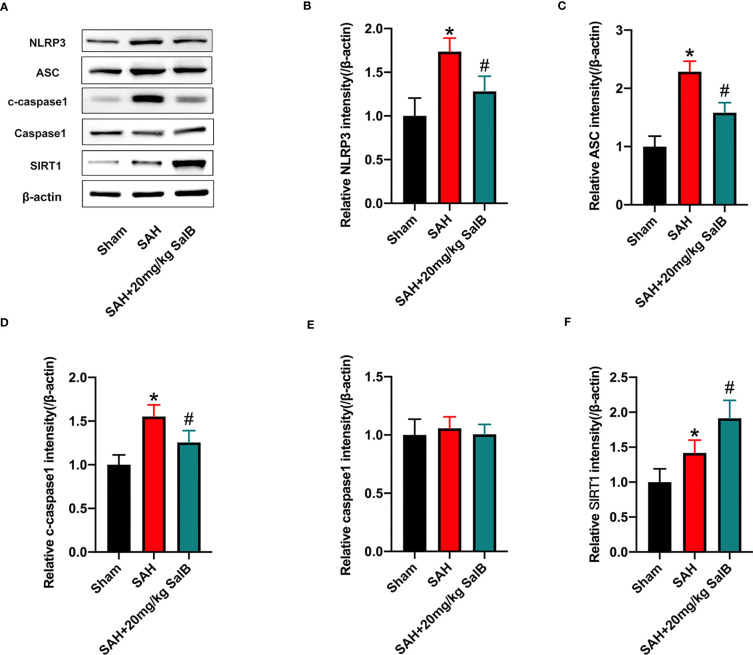
SalB inhibited NLRP3 inflammasome activation and promoted SIRT1 expression. **(A)** The protein expressions of NLRP3, ASC, caspase-1, c-caspase-1, and SIRT1 in ipsilateral cortex were detected by Western blot. Quantitative analysis of the levels of NLRP3 **(B)**, ASC **(C)**, c-caspase-1 **(D)**, caspase-1 **(E)**, and SIRT1 **(F)** in experimental groups (n = 6 mice/group). Data are expressed as mean ± SD. *P < 0.05 vs Sham group; ^#^ *P* < 0.05 vs SAH group.

Increasing evidence has implicated NLRP3 inflammasome as a main contributing factor in microglia-mediated inflammatory processes after SAH. Meanwhile, SIRT1 could inhibit NLRP3 inflammasome signaling (23, 24). We then evaluated whether SalB could regulate NLRP3 inflammasome and SIRT1 signaling to inhibit neuroinflammation. The NLRP3 inflammasome has three main components: the sensor protein NLRP3, the enzyme caspase 1, and the adaptor protein ASC. As shown in **Figure 3A**, western blotting results showed that the protein levels of NLRP3 (**Figure 3B**), ASC (**Figure 3C**), and cleaved-caspase1 (**Figure 3D**) were significantly increased after SAH when compared with the sham group, whereas SalB significantly reduced these proteins. No significant difference was detected in the expression of caspase1 among all experimental groups (**Figure 3E**). Additionally, the expression of SIRT1 was markedly increased after SAH, which was further enhanced by SalB administration (**Figure 3F**).

### SalB mitigated neuronal apoptosis after SAH

**Figure 4:**
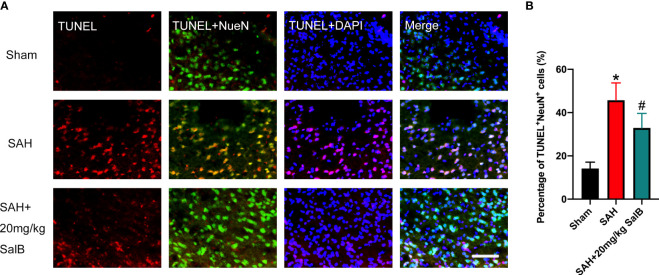
SalB mitigated neuronal apoptosis after SAH. **(A)** Representative images of TUNEL with NeuN (scale bar = 50 μm). **(B)** Quantitative analysis of the TUNEL^+^NeuN^+^ proportion in experimental groups (n = 6 mice/group). Data are expressed as mean ± SD. *P < 0.05 vs Sham group; ^#^ *P* < 0.05 vs SAH group.

Evidence has indicated that neuronal death is closely associated with poor neurological deficits after SAH. We then examined the effects of SalB on neuronal apoptosis. As shown in **Figure 4A**, the number of TUNEL^+^NeuN^+^ cells was significantly increased after SAH when compared with the sham group. In contrast, mice treated with SalB had remarkably lower apoptotic neurons than those in the SAH group (**Figure 4B**).

### Effects of EX527 and MCC950 on NLRP3 inflammasome and SIRT1 signaling

**Figure 5:**
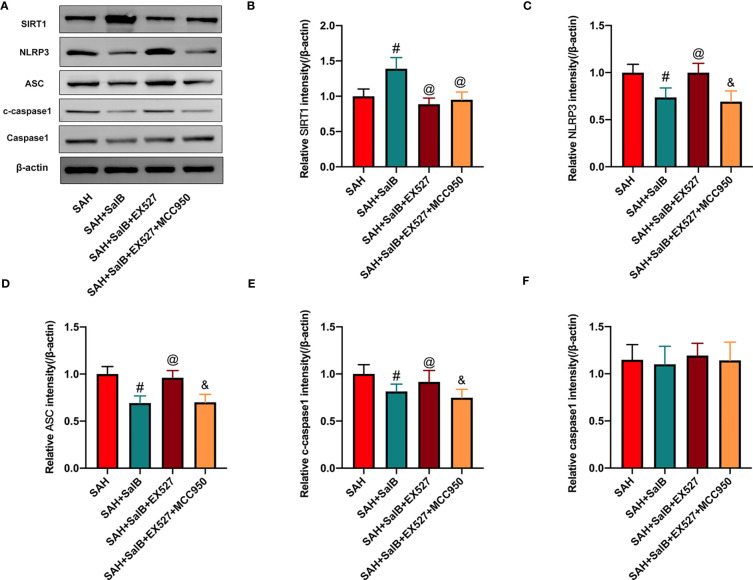
Effects of EX527 and MCC950 on NLRP3 inflammasome and SIRT1 signaling. **(A)** The protein expressions of SIRT1, NLRP3, ASC, caspase-1, and c-caspase-1 in ipsilateral cortex were detected by Western blot. Quantitative analysis of the levels of SIRT1 **(B)**, NLRP3 **(C)**, ASC **(D)**, c-caspase-1**(E)**, and caspase-1 **(F)** in experimental groups (n = 6 mice/group). Data are expressed as mean ± SD. ^#^ *P* < 0.05 vs SAH group, ^@^ *P* < 0.05 vs SAH + SalB group, ^&^ *P* < 0.05 vs SAH + SalB + EX527 group.

To validate the effect of SalB on NLRP3 inflammasome and SIRT1 signaling, EX527 and MCC950 were employed in this experiment. Consistent with a previous study (24), as shown in **Figure 5A**, our data showed that EX527 significantly reduced the increased SIRT1 expression induced by SalB (**Figure 5B**). Additionally, EX527 further induced NLRP3 inflammasome activation, as evidenced by the increased expression of NLRP3 (**Figure 5C**), ASC (**Figure 5D**), and cleaved-caspase1 (**Figure 5E**). No significant difference was detected in the expression of caspase1 among all experimental groups (**Figure 5F**). In contrast, the activated NLRP3 inflammasome signaling by EX527 could be suppressed by MCC950, as evidenced by the decreased expression of NLRP3 (**Figure 5C**), ASC (**Figure 5D**), and cleaved-caspase1 (**Figure 5E**). However, MCC950 did not affect SIRT1 expression after SAH (**Figure 5B**).

### Effects of EX527 and MCC950 on inflammatory response and microglial activation

**Figure 6:**
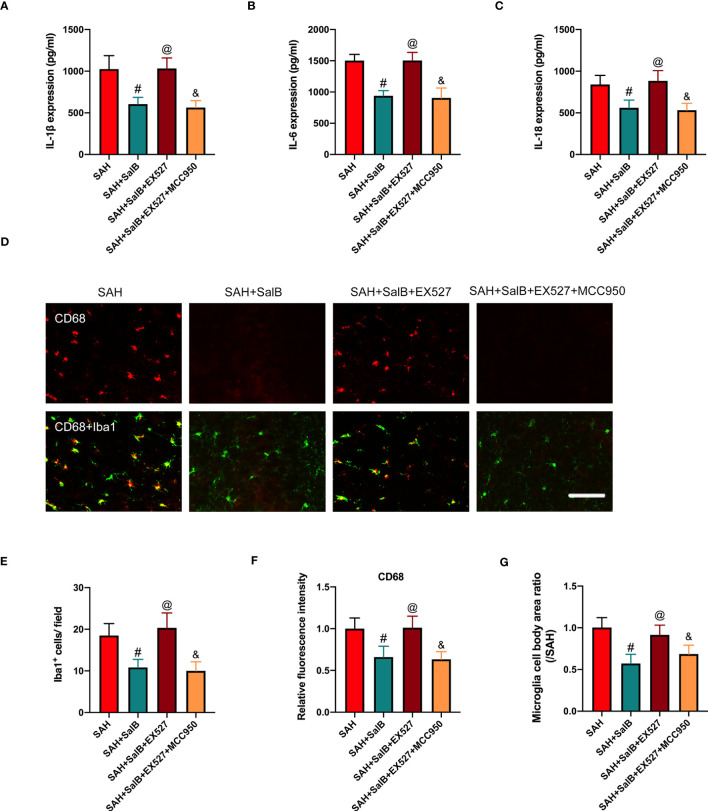
Effects of EX527 and MCC950 on inflammatory response and microglial activation. Quantitative analysis of the levels of IL-1β **(A)**, IL-6 **(B)**, and IL-18 **(C)** by ELISA kits (n = 6 mice/group). **(D)** Representative immunofluorescent staining of Iba1 with CD68 in ipsilateral cortex. Quantitative analysis of the number of Iba1^+^ cells **(E)**, CD68 intensity **(F)**, and microglia cell body area ratio **(G)** (n = 6 mice/group). Data are expressed as mean ± SD, scale bar = 50 μm. ^#^ *P* < 0.05 vs SAH group, ^@^ *P* < 0.05 vs SAH + SalB group, ^&^ *P* < 0.05 vs SAH + SalB + EX527 group.

Based on the findings above, we suspected that EX527 might further aggravate neuroinflammation. We then assessed the effects of EX527 and MCC950 on inflammatory response and microglial activation after SAH. As shown in **Figure 6**, EX527 treatment significantly reversed the anti-inflammatory effects of SalB against SAH, as evidenced by the increased levels of pro-inflammatory cytokines IL-1β (**Figure 6A**), IL-6 (**Figure 6B**), and IL-18 (**Figure 6C**). In addition, immunofluorescence staining (**Figure 6D**) showed that EX527 further increased the number of Iba1^+^ cells (**Figure 6E**), the immunoactivity of CD68 (**Figure 6F**), and microglia cell body area (**Figure 6G**). In contrast, all these changes induced by EX527 were reversed after treatment with MCC950 (**Figures 6A–G**). These suggested that inhibition SIRT1 by EX527 could aggravate neuroinflammation, and that suppression of NLRP3 by MCC950 might reduce neuroinflammation after SAH.

### Effects of EX527 and MCC950 on neuronal death and neurological behavior

**Figure 7:**
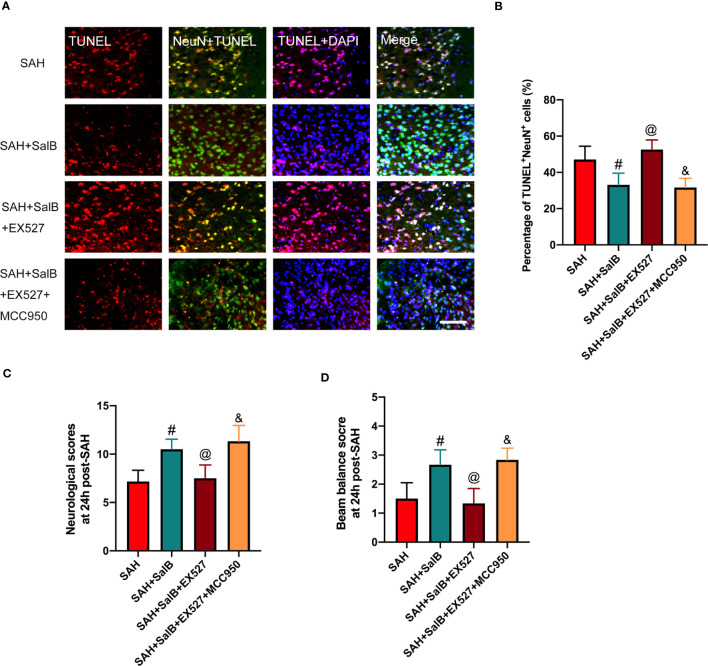
Effects of EX527 and MCC950 on neuronal death and neurological behavior. **(A)** Representative images of TUNEL with NeuN (scale bar = 50 μm). **(B)** Quantitative analysis of the TUNEL^+^NeuN^+^ proportion in experimental groups (n = 6 mice/group). **(C)** Neurological deficits test was performed on day 1 after SAH (n = 6 mice/group). **(D)** Beam balance test was conducted on day 1 after SAH (n = 6 mice/group). Data are expressed as mean ± SD. ^#^ *P* < 0.05 vs SAH group, ^@^ *P* < 0.05 vs SAH + SalB group, ^&^ *P* < 0.05 vs SAH + SalB + EX527 group.

In this experiment, we then explored the effects of EX527 and MCC950 on neuronal death and neurological behavior. As shown in **Figure 7A**, TUNEL staining indicated that EX527 treatment aggravated neuronal apoptotic index when compared with the SAH + SalB group (**Figure 7B**). In addition, EX527 treatment further exacerbated neurological deficits score (**Figure 7C**), and motor function (**Figure 7D**). In contrast, when compared with the SAH + SalB + EX527 group, MCC950 significantly improved neurological function and reduced neuronal death (**Figures 7A–D**). Based on the above results, these findings indicated that SalB could inhibit microglia-mediated inflammatory responses and prevent neuronal death after SAH through modulating NLRP3 inflammasome and SIRT1 signaling.

## Discussion

In this study, we performed an endovascular perforation SAH model to illuminate the potential anti-inflammatory functions of SalB on EBI after SAH and explored its molecular mechanisms. We found that SalB ameliorated SAH-induced functional behavior and cognitive deficits. Mechanistically, SalB mitigated microglial activation, pro-inflammatory cytokines release, and neuronal injury. Furthermore, SalB inhibited NLRP3 inflammasome activation and promoted SIRT1 expression after SAH. Administration of EX527 abrogated the anti-inflammatory effects of SalB against SAH and further induced NLRP3 inflammasome activation. In contrast, MCC950, a potent and selective NLRP3 inflammasome inhibitor, abolished the detrimental effects of SIRT1 inhibition by EX527 on EBI. These results indicated that SalB effectively repressed neuroinflammatory responses and neuronal damage after SAH by targeting NLRP3 inflammasome and SIRT1 signaling (**Supplementary Figure 2**).

Microglia-mediated neuroinflammation is involved in the pathology of EBI after SAH (2, 7). As the innate immune cells in CNS, microglia are early responder after SAH, which produce pro-inflammatory cytokines and cause damage to neuronal function and behavior function. Increasing evidence from both animal models and clinical studies has implicated the NLRP3 inflammasome as a main contributing factor in microglia-mediated inflammatory processes after SAH (23, 24, 28). The NLRP3 inflammasome is a cytosolic multi-protein complex that has three main components: the sensor protein NLRP3, the enzyme caspase 1, and the adaptor protein ASC. Upon stimulation, the NLRP3 inflammasome is assembled to trigger caspase-1 activation and subsequent pro-inflammatory cytokines release. Pharmacological inhibition of NLRP3 inflammasome is a promising therapeutic strategy for the treatment of SAH. SalB acts at multiple targets and has been shown to exert neuroprotective effects. In addition to its anti-oxidant property, SalB exhibits a potent anti-inflammatory activity and could inhibit microglial activation in a variety of CNS disorders (17, 29). Notably, studies have reported that SalB could repress NLRP3 inflammasome signaling (18, 19). Although SalB was reported to exhibit anti-inflammatory effects in many CNS disorders, it is unclear whether it targets microglia and NLRP3 inflammasome-mediated signaling after SAH.

Considering that microglia-mediated inflammatory response contributes greatly to EBI after SAH and the important role of NLRP3 inflammasome signaling in this response, we speculated that SalB might have a regulatory effect on microglial activation after SAH by inhibiting NLRP3 inflammasome. In agreement with previous reports (16, 17), SalB significantly inhibited microglial activation and decreased pro-inflammatory cytokines release after SAH. Concomitant with the decreased neuroinflammation, SalB improved behavior function and ameliorated cognitive impairment after SAH. These actions can be explained by the SalB-induced inhibition of NLRP3 inflammasome. However, how SalB regulates NLRP3 inflammasome activation after SAH remains unclear.

By searching the references, SalB is potent SIRT1 activator (30, 31). Meanwhile, a previous study reported that SalB could significantly mitigate SAH-induced oxidative damage by enhancing SIRT1 activation (9). Interestingly, we previously demonstrated that SIRT1 could inhibit NLRP3 inflammasome signaling to ameliorate EBI after SAH (32). As an important endogenous protective factor, SIRT1 could modulate a broad range of biological functions, including inflammation and oxidative stress (33, 34). Thus, we hypothesized that SalB might promote SIRT1 to inhibit NLRP3 inflammasome activation after SAH. Consistent with the previous study, we found that SalB increased SIRT1 expression, whereas EX527 suppressed SIRT1 activation and further induced NLRP3 inflammasome. Consistently, EX527 abated the anti-inflammatory effects of SalB against SAH. MCC950, a selective NLRP3 inflammasome inhibitor, has previously been shown to suppress NLRP3 inflammasome formation and neuroinflammation (35). Additionally, in animal models of traumatic brain injury and stroke, MCC950 could mitigate microglial activation, neurological deficits, brain edema, and neural death. These suggested that specific NLRP3 inflammasome inhibition using MCC950 might be an effective strategy for acute brain injuries. Therefore, we further employed MCC950 to validate the relationship between SIRT1 and NLRP3 inflammasome. In line with previous studies (36, 37), MCC950 treatment also repressed the activation of NLRP3 inflammasome and abated the detrimental effects of EX527 on EBI after SAH. In view of these results and the notion that microglia are the primary source of NLRP3 inflammasome, we postulated that SalB provided anti-inflammatory effects after SAH by inhibiting NLRP3 inflammasome and promoting SIRT1 pathway.

We noted that some scholars have reported that SalB exerts cerebroprotection effects after SAH by inhibiting oxidative damage (9). Different with this study, we performed an endovascular puncture SAH model and mainly studied the anti-inflammatory effects of SalB on microglia and NLRP3 inflammasome signaling. Although microglial response has detrimental effects in the early period after SAH, microglia also contribute to phagocytosis of cell debris and neural repair (38, 39). A previous study in major depressive disorder model suggested that SalB could promote microglial M2-polarization and rescue neurogenesis (17). However, whether SalB promoted M2 microglial polarization after SAH remains unclear. Additionally, it should be noted that many pharmacologic agents could target NLRP3 inflammasome and ameliorate EBI after SAH. However, few of them can be translated into clinical practice. The possible explanation might be lacking enough toxicological studies. Although no side-effects of SalB were reported, the toxicological studies of SalB and its optimal and safe dose still need to be investigated. Finally, we cannot exclude the possibility that other molecular targets might involve in the anti-inflammatory effects of SalB after SAH.

## Conclusions

In summary, we discovered that SalB inhibited microglia-mediated inflammatory responses and prevented neuronal damage after SAH through modulating NLRP3 inflammasome and SIRT1 signaling.

## Data availability statement

The raw data supporting the conclusions of this article will be made available by the authors upon reasonable request.

## Ethics statement

All procedures were approved by the Animal Care and Use Committee of Yijishan Hospital.

## Author contributions

DX, HD, and ZZ conceived the research. DX, JY, DW, HD, and ZZ performed the experiments; DX and HD analyzed the results; DX and ZZ wrote and revised the manuscript. All authors contributed to the article and approved the submitted version.

## Supplementary material

Supplementary Figure 1 Schematic illustration of experiment design. In the first set of experiments, the effects of SalB on short-term and long-term functional behavior after SAH were evaluated. In the second experiment, the potential role of SalB on neuroinflammation and its mechanisms were evaluated. In the third experiment, the effects of SalB on NLRP3 inflammasome and SIRT1 signaling were validated by using EX527 and MCC950.

Supplementary Figure 2 As innate immune cells in the CNS, microglia have been implicated as a primary factor in inflammation-mediated brain damage. After SAH, NLRP3 inflammasome activation plays a crucial role in microglia-mediated inflammatory responses. The NLRP3 inflammasome is assembled to trigger caspase-1 activation and subsequent pro-inflammatory cytokines release. Inhibition of NLRP3 inflammasome activation by MCC950 could mitigate microglia activation and neuroinflammation after SAH. In addition, SIRT1 activation could inhibit NLRP3 inflammasome to reduce brain damage after SAH. SalB treatment promotes SIRT1 activation and inhibits NLRP3 inflammasome, thereby ameliorating neuronal damage after SAH. In contrast, SIRT1 inhibition by EX527 reverses the protective effects of SalB against SAH.

## References

[1] Macdonald RL Schweizer TA. Spontaneous subarachnoid haemorrhage. Lancet (2017) 389:655–66. doi: 10.1016/S0140-6736(16)30668-7 27637674

[2] Zeyu Z Yuanjian F Cameron L Sheng C. The role of immune inflammation in aneurysmal subarachnoid hemorrhage. Exp Neurol (2021) 336:113535. doi: 10.1016/j.expneurol.2020.113535 33249033

[3] Weiland J Beez A Westermaier T Kunze E Siren AL Lilla N. Neuroprotective strategies in aneurysmal subarachnoid hemorrhage (asah). Int J Mol Sci (2021) 22. doi: 10.3390/ijms22115442 PMC8196706 34064048

[4] Dodd WS Laurent D Dumont AS Hasan DM Jabbour PM Starke RM. Pathophysiology of delayed cerebral ischemia after subarachnoid hemorrhage: A review. J Am Heart Assoc (2021) 10:e021845. doi: 10.1161/JAHA.121.021845 34325514 PMC8475656

[5] Qu W Cheng Y Peng W Wu Y Rui T Luo C. Targeting inos alleviates early brain injury after experimental subarachnoid hemorrhage via promoting ferroptosis of m1 microglia and reducing neuroinflammation. Mol Neurobiol (2022) 59:3124–39. doi: 10.1007/s12035-022-02788-5 35262869

[6] Dodd WS Noda I Martinez M Hosaka K Hoh BL. Nlrp3 inhibition attenuates early brain injury and delayed cerebral vasospasm after subarachnoid hemorrhage. J Neuroinflamm (2021) 18:163. doi: 10.1186/s12974-021-02207-x PMC8293512 34284798

[7] Savarraj J Parsha K Hergenroeder G Ahn S Chang TR Kim DH. Early brain injury associated with systemic inflammation after subarachnoid hemorrhage. Neurocrit Care (2018) 28:203–11. doi: 10.1007/s12028-017-0471-y 29043545

[8] Deng HJ Deji Q Zhaba W Liu JQ Gao SQ Han YL. A20 establishes negative feedback with traf6/nf-kappab and attenuates early brain injury after experimental subarachnoid hemorrhage. Front Immunol (2021) 12:623256. doi: 10.3389/fimmu.2021.623256 34381441 PMC8350325

[9] Zhang X Wu Q Lu Y Wan J Dai H Zhou X. Cerebroprotection by salvianolic acid b after experimental subarachnoid hemorrhage occurs via nrf2- and sirt1-dependent pathways. Free Radic Biol Med (2018) 124:504–16. doi: 10.1016/j.freeradbiomed.2018.06.035 PMC6286712 29966698

[10] Zhao XS Zheng B Wen Y Sun Y Wen JK Zhang XH. Salvianolic acid b inhibits ang ii-induced vsmc proliferation *in vitro* and intimal hyperplasia *in vivo* by downregulating mir-146a expression. Phytomedicine (2019) 58:152754. doi: 10.1016/j.phymed.2018.11.014 31009837

[11] Zhao R Liu X Zhang L Yang H Zhang Q. Current progress of research on neurodegenerative diseases of salvianolic acid b. Oxid Med Cell Longev (2019) 2019:3281260. doi: 10.1155/2019/3281260 31341529 PMC6612994

[12] Katary MA Abdelsayed R Alhashim A Abdelhasib M Elmarakby AA. Salvianolic acid b slows the progression of breast cancer cell growth via enhancement of apoptosis and reduction of oxidative stress, inflammation, and angiogenesis. Int J Mol Sci (2019) 20. doi: 10.3390/ijms20225653 PMC6888679 31726654

[13] Chen T Liu W Chao X Zhang L Qu Y Huo J. Salvianolic acid b attenuates brain damage and inflammation after traumatic brain injury in mice. Brain Res Bull (2011) 84:163–8. doi: 10.1016/j.brainresbull.2010.11.015 21134421

[14] Zhu Z Ding L Qiu WF Wu HF Li R. Salvianolic acid b protects the myelin sheath around injured spinal cord axons. Neural Regener Res (2016) 11:487–92. doi: 10.4103/1673-5374.179068 PMC4829017 27127491

[15] Habtemariam S. Molecular pharmacology of rosmarinic and salvianolic acids: Potential seeds for alzheimer's and vascular dementia drugs. Int J Mol Sci (2018) 19:458. doi: 10.3390/ijms19020458 29401682 PMC5855680

[16] Wu JZ Ardah M Haikal C Svanbergsson A Diepenbroek M Vaikath NN. Dihydromyricetin and salvianolic acid b inhibit alpha-synuclein aggregation and enhance chaperone-mediated autophagy. Transl Neurodegener (2019) 8:18. doi: 10.1186/s40035-019-0159-7 31223479 PMC6570948

[17] Zhang J Xie X Tang M Zhang J Zhang B Zhao Q. Salvianolic acid b promotes microglial m2-polarization and rescues neurogenesis in stress-exposed mice. Brain Behav Immun (2017) 66:111–24. doi: 10.1016/j.bbi.2017.07.012 28736034

[18] Tang Y Wa Q Peng L Zheng Y Chen J Chen X. Salvianolic acid b suppresses er stress-induced nlrp3 inflammasome and pyroptosis via the ampk/foxo4 and syndecan-4/rac1 signaling pathways in human endothelial progenitor cells. Oxid Med Cell Longev (2022) 2022:8332825. doi: 10.1155/2022/8332825 35340217 PMC8947883

[19] Li Q Zuo Z Pan Y Zhang Q Xu L Jiang B. Salvianolic acid b alleviates myocardial ischemia injury by suppressing nlrp3 inflammasome activation via sirt1-ampk-pgc-1alpha signaling pathway. Cardiovasc Toxicol (2022) 22:842–57. doi: 10.1007/s12012-022-09760-8 35809215

[20] Walsh JG Muruve DA Power C. Inflammasomes in the cns. Nat Rev Neurosci (2014) 15:84–97. doi: 10.1038/nrn3638 24399084

[21] Denes A Lopez-Castejon G Brough D. Caspase-1: Is il-1 just the tip of the iceberg? Cell Death Dis (2012) 3:e338. doi: 10.1038/cddis.2012.86 22764097 PMC3406585

[22] Miao EA Rajan JV Aderem A. Caspase-1-induced pyroptotic cell death. Immunol Rev (2011) 243:206–14. doi: 10.1111/j.1600-065X.2011.01044.x PMC3609431 21884178

[23] Hu X Yan J Huang L Araujo C Peng J Gao L. Int-777 attenuates nlrp3-asc inflammasome-mediated neuroinflammation via tgr5/camp/pka signaling pathway after subarachnoid hemorrhage in rats. Brain Behav Immun (2021) 91:587–600. doi: 10.1016/j.bbi.2020.09.016 32961266 PMC7749833

[24] Zhang XS Lu Y Li W Tao T Wang WH Gao S. Cerebroprotection by dioscin after experimental subarachnoid haemorrhage via inhibiting nlrp3 inflammasome through sirt1-dependent pathway. Br J Pharmacol (2021) 178:3648–66. doi: 10.1111/bph.15507 33904167

[25] Tao T Liu GJ Shi X Zhou Y Lu Y Gao YY. Dhea attenuates microglial activation via induction of jmjd3 in experimental subarachnoid haemorrhage. J Neuroinflamm (2019) 16:243. doi: 10.1186/s12974-019-1641-y PMC6883548 31779639

[26] Xu P Tao C Zhu Y Wang G Kong L Li W. Tak1 mediates neuronal pyroptosis in early brain injury after subarachnoid hemorrhage. J Neuroinflamm (2021) 18:188. doi: 10.1186/s12974-021-02226-8 PMC8406585 34461942

[27] Clarke JV Brier LM Rahn RM Diwan D Yuan JY Bice AR. Sirt1 mediates hypoxic postconditioning- and resveratrol-induced protection against functional connectivity deficits after subarachnoid hemorrhage. J Cereb Blood Flow Metab (2022) 42:1210–23. doi: 10.1177/0271678X221079902 PMC9207494 35137611

[28] Xu P Hong Y Xie Y Yuan K Li J Sun R. Trem-1 exacerbates neuroinflammatory injury via nlrp3 inflammasome-mediated pyroptosis in experimental subarachnoid hemorrhage. Transl Stroke Res (2021) 12:643–59. doi: 10.1007/s12975-020-00840-x 32862402

[29] Yin X Feng L Ma D Yin P Wang X Hou S. Roles of astrocytic connexin-43, hemichannels, and gap junctions in oxygen-glucose deprivation/reperfusion injury induced neuroinflammation and the possible regulatory mechanisms of salvianolic acid b and carbenoxolone. J Neuroinflamm (2018) 15:97. doi: 10.1186/s12974-018-1127-3 PMC5872583 29587860

[30] Guo Y Yang JH Cao SD Gao CX He Y Wang Y. Effect of main ingredients of danhong injection against oxidative stress induced autophagy injury via mir-19a/sirt1 pathway in endothelial cells. Phytomedicine (2021) 83:153480. doi: 10.1016/j.phymed.2021.153480 33548866

[31] He Y Lu R Wu J Pang Y Li J Chen J. Salvianolic acid b attenuates epithelial-mesenchymal transition in renal fibrosis rats through activating sirt1-mediated autophagy. BioMed Pharmacother (2020) 128:110241. doi: 10.1016/j.biopha.2020.110241 32450523

[32] Xia DY Yuan JL Jiang XC Qi M Lai NS Wu LY. Sirt1 promotes m2 microglia polarization via reducing ros-mediated nlrp3 inflammasome signaling after subarachnoid hemorrhage. Front Immunol (2021) 12:770744. doi: 10.3389/fimmu.2021.770744 34899720 PMC8653696

[33] Han X Ding C Sang X Peng M Yang Q Ning Y. Targeting sirtuin1 to treat aging-related tissue fibrosis: From prevention to therapy. Pharmacol Ther (2022) 229:107983. doi: 10.1016/j.pharmthera.2021.107983 34480962

[34] Vellimana AK Diwan D Clarke J Gidday JM Zipfel GJ. Sirt1 activation: A potential strategy for harnessing endogenous protection against delayed cerebral ischemia after subarachnoid hemorrhage. Neurosurgery (2018) 65:1–5. doi: 10.1093/neuros/nyy201 31076789 PMC8892594

[35] Xu X Yin D Ren H Gao W Li F Sun D. Selective nlrp3 inflammasome inhibitor reduces neuroinflammation and improves long-term neurological outcomes in a murine model of traumatic brain injury. Neurobiol Dis (2018) 117:15–27. doi: 10.1016/j.nbd.2018.05.016 29859317

[36] Palomino-Antolin A Narros-Fernandez P Farre-Alins V Sevilla-Montero J Decouty-Perez C Lopez-Rodriguez AB. Time-dependent dual effect of nlrp3 inflammasome in brain ischaemia. Br J Pharmacol (2022) 179:1395–410. doi: 10.1111/bph.15732 34773639

[37] Corcoran SE Halai R Cooper MA. Pharmacological inhibition of the nod-like receptor family pyrin domain containing 3 inflammasome with mcc950. Pharmacol Rev (2021) 73:968–1000. doi: 10.1124/pharmrev.120.000171 34117094

[38] Yang LY Chen YR Lee JE Chen KW Luh HT Chen YT. Dental pulp stem cell-derived conditioned medium alleviates subarachnoid hemorrhage-induced microcirculation impairment by promoting m2 microglia polarization and reducing astrocyte swelling. Transl Stroke Res (2022) 10. doi: 10.1007/s12975-022-01083-8 PMC10444696 36181630

[39] Tao T Chen X Zhou Y Zheng Q Gao S Wang J. Continued p2x7 activation leads to mitochondrial fission and compromising microglial phagocytosis after subarachnoid haemorrhage. J Neurochem (2022) 163:419–37. doi: 10.1111/jnc.15712 PMC9828135 36269673

