# Supplementary figures and images for: Salvianolic acid B ameliorates neuroinflammation and neuronal injury via blocking NLRP3 inflammasome and promoting SIRT1 in experimental subarachnoid hemorrhage
Source: Front Immunol. 2023 Jul 26;14:1159958. doi: 10.3389/fimmu.2023.1159958 (PMC10410262; doi:10.3389/fimmu.2023.1159958)

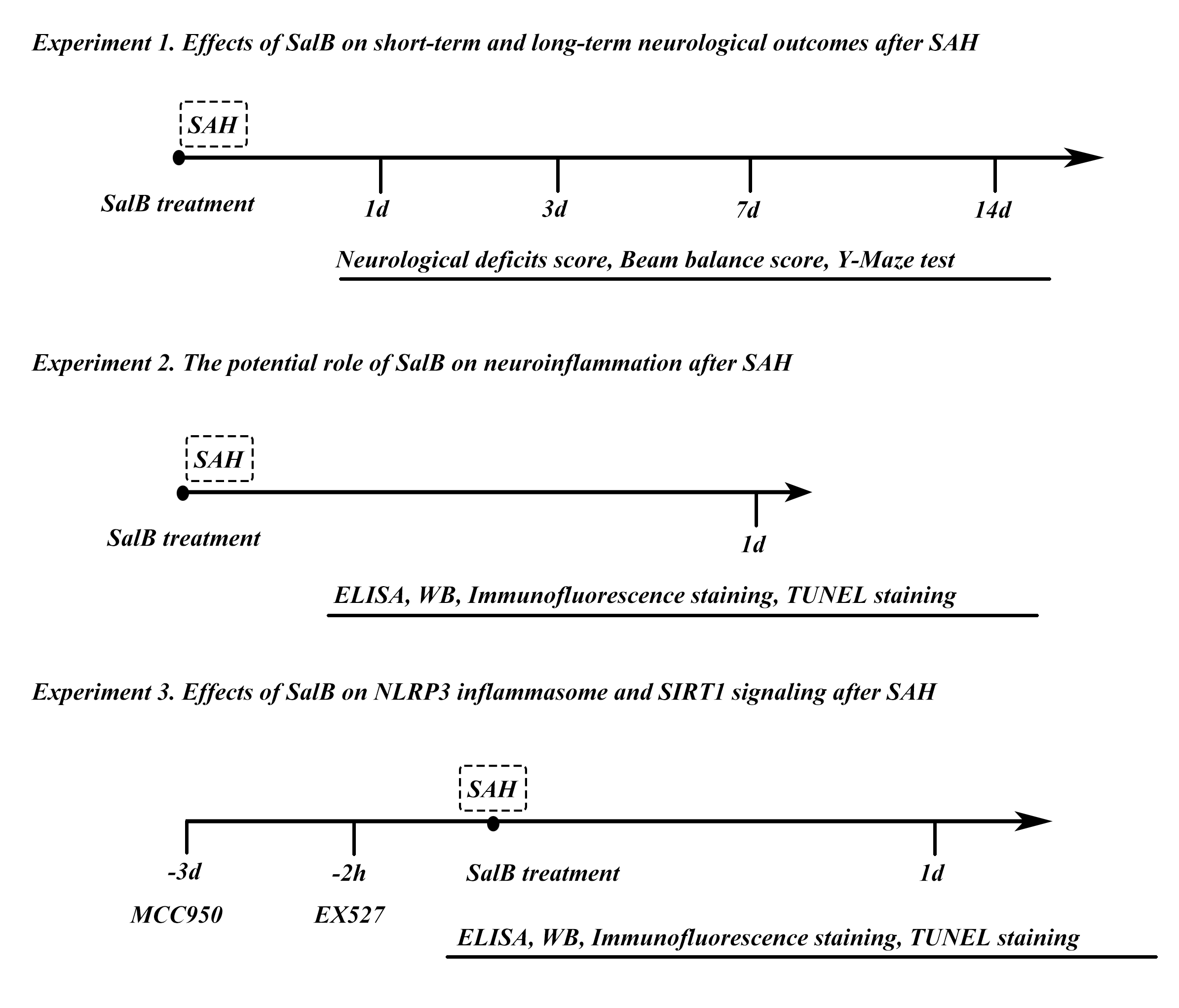

Supplement: Supplementary Figure 1 — Schematic illustration of experiment design. In the first set of experiments, the effects of SalB on short-term and long-term functional behavior after SAH were evaluated. In the second experiment, the potential role of SalB on neuroinflammation and its mechanisms were evaluated. In the third experiment, the effects of SalB on NLRP3 inflammasome and SIRT1 signaling were validated by using EX527 and MCC950. [file Image_1.tif]

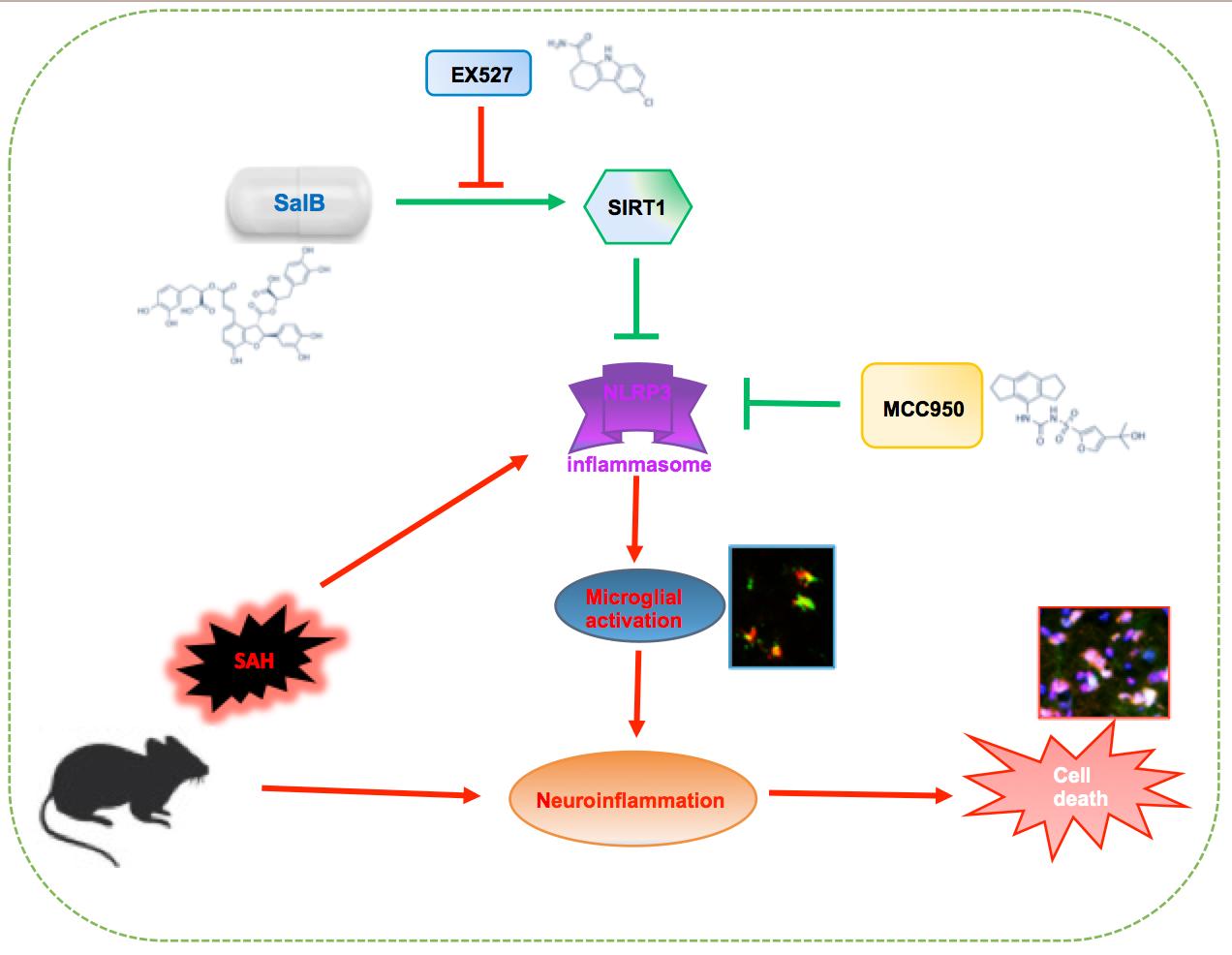

Supplement: Supplementary Figure 2 — As innate immune cells in the CNS, microglia have been implicated as a primary factor in inflammation-mediated brain damage. After SAH, NLRP3 inflammasome activation plays a crucial role in microglia-mediated inflammatory responses. The NLRP3 inflammasome is assembled to trigger caspase-1 activation and subsequent pro-inflammatory cytokines release. Inhibition of NLRP3 inflammasome activation by MCC950 could mitigate microglia activation and neuroinflammation after SAH. In addition, SIRT1 activation could inhibit NLRP3 inflammasome to reduce brain damage after SAH. SalB treatment promotes SIRT1 activation and inhibits NLRP3 inflammasome, thereby ameliorating neuronal damage after SAH. In contrast, SIRT1 inhibition by EX527 reverses the protective effects of SalB against SAH. [file Image_2.tif]
